# Analysis of Parameters of a Rectified Tank on the Basis of In-Situ Tests

**DOI:** 10.3390/ma14143881

**Published:** 2021-07-12

**Authors:** Krzysztof Gromysz

**Affiliations:** Department of Building Structures, Faculty of Civil Engineering, Silesian University of Technology, 44-100 Gliwice, Poland; krzysztof.gromysz@polsl.pl

**Keywords:** support stiffness, parameters of the structure, stiffness matrix, form of displacement, vertical deflection of building structure, rectification, work done on moving building objects

## Abstract

The vertical deflection of building structures is a common problem. However, the rectification of objects is rarely carried out due to the lack of information about the parameters of objects requiring rectification. The subject of the analysis are parameters of rectified water tank 950 m^3^ in volume, which were investigated due to the stiffness and number of supports built into the structure. During in-situ testing, the stiffnesses of supports were determined. The model of the rectified structure was then defined and it was shown that its parameters can be described by means of three matrices: stiffness, displacement forms of the elevated object and displacement forms of supports. Absolute values of elements of the stiffness matrix increased as the stiffness and number of supports increased. At the same time, values of elements of the matrix of displacement forms of the elevated object increased. The conducted energy analysis demonstrated that the amount of energy required for the vertical displacement of the structure decreased with an increasing stiffness and number of supports. This means that placing a greater number of supports under rectified structures and ensuring more rigid supports is beneficial to the rectification. Results of the conducted analyses were confirmed during in-situ tests.

## 1. Introduction

Vertical deflection is a common defect of building structures. This effect is mainly caused by insufficient load-bearing capacity of subsoil [1,2]. This problem concerns different types of construction: grain elevators [3], historic masonry [4] and wood towers [5,6], churches [7], and residential buildings [8]. Deflection of building objects can be also caused by earthquakes [9], wind loading [10] and the construction of tunnels in their vicinity [11]. Moreover, many buildings are deflected as the effect of non-uniform subsidence of mining areas [12], resulting from the compression of voids left after coal bed extraction [13]. Subsidence of the mining area may take the form of continuous [14,15] or discontinuous deformation [16]. Deflections can be also caused by exploitation of groundwater reservoirs [17]. However, deflection increases the seismic vulnerability of towers [18], slender structures and tall buildings [19]. Moreover, deflection, an effect of nonuniform settlement of the ground, can damage constructions with wall building structure [20,21], frame structures [22], foundations [23] which can increase the risk of building operation [24], and the risk of collapse [25]. Deflected structures are usually stabilized by reinforcing their foundation with steel [26] or reinforced concrete piles [27], or by reinforcing the ground [4]. In case of the deflection of single elements of the structure, they are stabilised in a new position [28]. In particular situations, structural elements [29] or whole buildings [30] are rectified. This method can be based on removing soil from parts of the building that are too high [31,32,33] or elevating parts of the building that are too low [34] through the use of temporary supports [35]. The previous tests included the rectification of buildings which were divided into two parts, one of them remaining in the ground, while the other was non-uniformly elevated. Such a process with reference to historic two-storey building is described in [36]. No tests have been performed so far on parameters of rectified structures that are elevated in a non-uniform way with the foundation slab.

Hence, this paper defines a model of structure elevated in a non-uniform way with the foundation slab. Model parameters are determined for fire fighting storage tank of 950 m^3^ in volume. This structure was built on human-altered soil which caused its deflection.

**Figure 1:**
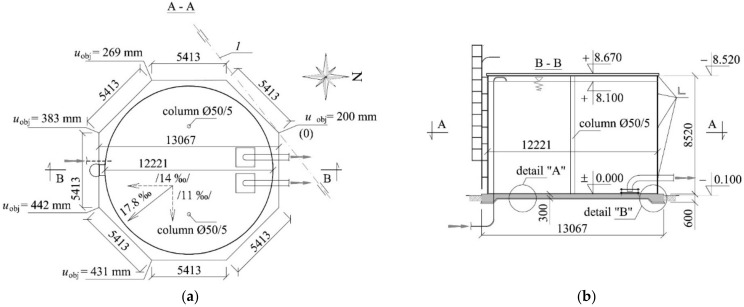
Tested steel water tank placed on the reinforced concrete foundation slab: (**a**) tank plan; (**b**) cross-section, 1—centre of rotation during rectification, 17.8‰—resultant deflection, /14%/, /11%/—components of deflection.

The steel tank has a cylindrical shape with an inner diameter of 12.221 m and a height of 8.520 m (Figure 1). Its shell is made of a metal sheet of 3 mm in thickness and reinforced with elements made of an angle section placed at four levels. The roof structure is composed of steel beams made of cold-formed steel elements supported on the reinforced edge of the shell and two columns placed inside the tank. A three-layer panel with polyurethane core is used as the roofing material. Inside the tank, there are water supply systems and components of the fire-fighting system used to pump out water. Waterproofing is ensured by PVC film of 1.5 mm in thickness which is placed inside the tank.

**Figure 2:**
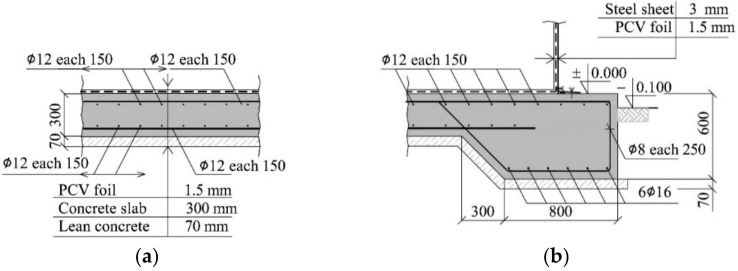
Reinforced concrete foundation slab of the tank: (**a**) detail “A” from Figure 1; (**b**) detail “B” from Figure 1.

The tank is placed on a reinforced concrete foundation slab of octagonal shape and a side length equal to 5.413 m. The slab has a thickness of 300 mm (Figure 2a). However, the thickness changes gently at the edges up to 600 mm and the reinforced concrete beam formed around the slab has a cross-section of (b/h) 800 mm/600 mm (Figure 2b). A layer of lean concrete of 70 mm in thickness is placed under the slab. The 2-way reinforcement of upper and bottom slabs is composed of rebars of 12 mm in diameter and the spacing of 150 mm. The bottom reinforcement of the beam around the slab contains six rebars having a diameter of 16 mm and stirrups made of bars of 8 mm in diameter with the spacing of 250 mm. The weight of an empty tank with the foundation slab is 1347 kN.

The tank was deflected by 17.8 mm/m from vertical in the south-east direction. Consequently, it required the rectification. For that purpose 16 stacks of concrete blocks were pressed into the ground under reinforced concrete slab. Then, 16 hydraulic jacks were placed on these jack supports. These jacks took the weight of the tank. They were used to elevate uniformly the tank and the foundation by 200 mm, and then the rectification began by non-uniform elevation. It means the whole tank was rotated around axis 1 (Figure 1). The minimum elevation *u*_obj_ was 200 mm, and the maximum 431 mm (Figure 1). Space between the slab base and the ground formed as the result of rectification was filled with concrete.

In-situ testing of the structure placed on the hydraulic jacks was performed before and after the rectification. The results were used to determine stiffness of jack and jack supports and to determine the parameters of the rectified tank.

It should be noted that no tests have been performed so far on parameters of tanks that are elevated with the foundation slab in a non-uniform way. Similarly, no research into the lifting of whole building objects have been undertaken in the literature.

## 2. Parameters of Rectified Tank

**Figure 3:**
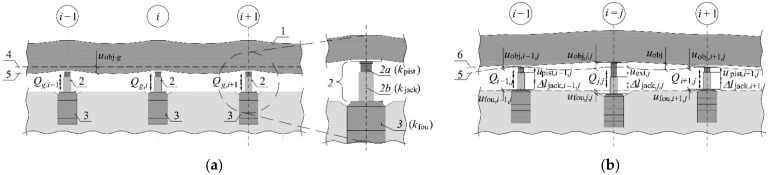
Displacements of the structure components, piston extensions and changes in forces in the jacks: (**a**) tank resting on the jacks prior to lifting; (**b**) tank after forced piston extension in the active *j*-th jack of value *u*_ext,*j*_: 1—elevated tank, 2—jack (2a—piston characterized by stiffness *k*_pist_, 2b—cylinder characterized by stiffness *k*_jack_), 3—jack support characterized by stiffness *k*_fou_, 4—position of the tank before installing the jacks, 5—position of the tank when jacks took the tank weight, 6—position of the elevated tank after the forced piston extension by the value *u*_ext,*j*_.

To define parameters of the rectified tank resting on the hydraulic jacks, the following elements were assumed to be elements of the tested system (Figure 3a): elevated tank (1), hydraulic jacks (2) and jack supports (3). Pistons (2a) and cylinders (2b) were considered to be exposed to independent extensions and deformations.

When the jacks took the tank weight, in each of them was generated the force *Q*_g,*i*_ whose value resulted from dead load and stiffness of individual components of the structure. Additionally, as the tank was placed on the jacks, it was deformed because of the acting dead load. The bottom edge of the slab changed its position of *u*_obj-*g*_ value from (4) to (5) (Figure 3a). Each *i*-th support for the jack was also displaced.

### Passive and Active Jacks

Each of *n* jacks, on which the tank is placed, can operate as active or passive jack during lifting. The piston forced extension by the value *u*_ext,*j*_ was forced in the active *j*-th jack (Figure 3b). This extension resulted in the displacements *u*_obj_ of the structure with reference to the position *u*_obj-*g*_, the piston extension in the adjacent jacks, displacement of jack supports, changes in the length of cylinders, and changes in values of forces in jacks.

The dual *i*,*j* indices specifying the effect of the forced piston extension in the *j*-th jack were assumed. The index *i* means a point number of the structure and the jack number, that effects are specified for the position of its installation. The index *j* corresponds to the number of the active jack, whose piston extension is forced. Each jack can be active or passive. Thus, the indices *i* and *j* can take values 1, 2, …, *n*, where *n* is the number of jacks installed under the tank. When the index specifying a given value is *i* = *j*, then it refers to changes in the position of the jack which is active at that moment.

Value *u*_ext,*j*_ of the forced piston extension in the *j*-th active jack (*i* = *j*) generated an increase in the force by *Q_j,j_* and resulted in the following changes at the point of the installed jack (Figure 3b):

- Upward displacement of value *u*_obj*,j,j*_ of the elevated structure;
- Downward displacement of value *u*_obj*,j,j*_ of the jack support;
- Change ∆*l*jack*,j,j* of the jack cylinder length.

The relationship between a change in the force value *Q_j,j_* of the jack and the forced piston extension *u*_ext*,j*_ (*i* = *j*) was assumed to be linear

(1) $$k_{j,j}=\frac{Q_{j,j}}{u_{\mathrm{ext},j}},\;$$

where *k_j,j_* is the structure stiffness at the point of forced displacement *u*_ext,*j*_.

The forced piston extension in the *j*-th active jack changed the force in the *i*-th passive jack of value *Q_i_*_,*j*_, caused the free piston extension *u*_pist,*i,j*_ of this jack and caused the following effects at the point of the installed *i*-th passive jack (Figure 3b):

- Upward displacement of value *u*_obj,*i,j*_ of the elevated part;
- Downward displacement of value *u*_fou,*i,j*_ of the jack support;
- Change ∆*l*_jack,*i,j*_ of the jack cylinder length.

The relationship between a change in *Q_i,j_* and *u*_ext,*j*_ was assumed to be linear

(2) $$k_{i,j}=\frac{Q_{i,j}}{u_{\mathrm{ext},j}},\;$$

where *k_i,j_* is a change in the force of the *i*-th passive jack caused by the forced piston extension *u*_ext,*j*_ equal to 1 in the *j*-th jack.

The jacks placed on the jack supports functioned as one-sided supports that did not transfer tensile forces. However, no pull-off was observed in these supports which was a consequence of high values *Q*_g,*i*_ resulting from the tank weight and low values *u*_ext,*j*_ of the forced piston extension. Therefore, the jack support model was assumed to be an element having the constant stiffness both at increasing and decreasing force in the support. The jack support, jack cylinder and its piston had the following stiffness values *k*_fou_, *k*_jack_, *k*_pist_, which were defined as

(3) $$k_{\mathrm{fou}}=\frac{Q_{i,j}}{u_{\mathrm{fou},i,j}},\;k_{\mathrm{jack}}=\frac{Q_{i,j}}{u_{\mathrm{jack},i,j}},\;k_{\mathrm{pist}}=\frac{Q_{i,j}}{u_{\mathrm{pist},i,j}}.$$

On the basis of the linear relationship between the forced extension *u*_ext,*j*_ of the piston and any displacement of the structure caused by this extension, the following constants for the structure were assumed

(4) $$f_{\mathrm{obj},i,j}=\frac{u_{\mathrm{obj},i,j}}{u_{\mathrm{ext},j}},$$

(5) $$f_{\mathrm{fou},i,j}=\frac{u_{\mathrm{fou},i,j}}{u_{\mathrm{ext},j}},$$

which define displacements of the elevated structure and the jack support caused by forced piston extension *u*_ext,j_ equal to 1.2.2. Description of Rectified Tank Expressed by Matrices

Changes in values of jack forces resulting from any forced piston extension can be expressed as:

(6) $$Q=ku_{\mathrm{ext}}$$

where

(7) $$Q=\left[\begin{array}{c} \begin{array}{c} Q_{1}\\ Q_{2}\\ \ldots \end{array}\\ \begin{array}{c} Q_{i}\\ \ldots \\ Q_{n} \end{array} \end{array}\right],\;\;u_{\mathrm{ext}}=\left[\begin{array}{c} \begin{array}{c} u_{\mathrm{ext},1}\\ u_{\mathrm{ext},2}\\ \ldots \end{array}\\ \begin{array}{c} u_{\mathrm{ext},j}\\ \ldots \\ u_{\mathrm{ext},n} \end{array} \end{array}\right]$$

are vectors of changes in jack forces and forced piston extensions, and **k** is the stiffness matrix

(8) $$k=\left[\begin{array}{cc} \begin{array}{ccc} k_{1,1} & k_{1,2} & \ldots \\ k_{2,1} & k_{2,2} & \ldots \\ \ldots & \ldots & \ldots \end{array} & \begin{array}{ccc} k_{1,j} & \ldots & k_{1,n}\\ k_{2,j} & \ldots & k_{2,n}\\ \ldots & \ldots & \ldots \end{array}\\ \begin{array}{ccc} k_{i,1} & k_{i,2} & \ldots \\ \ldots & \ldots & \ldots \\ k_{n,1} & k_{n,2} & \ldots \end{array} & \begin{array}{ccc} k_{i,j} & \ldots & k_{i,n}\\ \ldots & \ldots & \ldots \\ k_{n,j} & \ldots & k_{n,n} \end{array} \end{array}\right]$$

of the rectified tank placed on jacks, where *k_j,j_* (*i* = *j*), calculated from (1) and *k_i_*_,*j*_ (*i* ≠ *j*) calculated from (2).

The forced piston extension in the *j*-th jack cause displacements of elevated tank and jack supports. It was assumed that those displacements are represented by two vectors denoted as vectors of displacement forms of the elevated object and of the jack supports

(9) $$f_{\mathrm{obj},j}=\left[\begin{array}{c} \begin{array}{c} f_{\mathrm{obj},1,j}\\ f_{\mathrm{obj},2,j} \end{array}\\ \begin{array}{c} \ldots \\ f_{\mathrm{obj},i,j} \end{array}\\ \begin{array}{c} \ldots \\ f_{\mathrm{obj},n,j} \end{array} \end{array}\right],\;f_{\mathrm{fou},j}=\left[\begin{array}{c} \begin{array}{c} f_{\mathrm{fou},1,j}\\ f_{\mathrm{fou},2,j} \end{array}\\ \begin{array}{c} \ldots \\ f_{\mathrm{fou},i,j} \end{array}\\ \begin{array}{c} \ldots \\ f_{\mathrm{fou},n,j} \end{array} \end{array}\right],$$

where *f*_obj,*i*,*j*_ was calculated from (4), and *f*_fou,*i*,*j*_ was calculated from (5). Those vectors define with accuracy to the constant factor, the distribution of displacements of the elevated object and of the jack supports. The vector ***f***_obj,*j*_ was used to denote the *j*-th form of displacements of the elevated tank, and ***f***_fou,*j*_ was the *j*-th form of displacements of jack supports. 

A set of vectors ***f***_obj,*j*_ formed a matrix of displacement forms of the elevated part

(10) $$f_{\mathrm{obj}}=\left[\begin{array}{ccc} \begin{array}{cc} f_{\mathrm{obj},1} & f_{\mathrm{obj},2} \end{array} & \begin{array}{ccc} \ldots & f_{\mathrm{obj},j} & \ldots \end{array} & f_{\mathrm{obj},n} \end{array}\right]$$

and a set of vectors ***f***_fou,*j*_ formed a matrix of displacement forms of the jack supports

(11) $$f_{\mathrm{fou}}=\left[\begin{array}{ccc} \begin{array}{cc} f_{\mathrm{fou},1} & f_{\mathrm{fou},2} \end{array} & \begin{array}{ccc} \ldots & f_{\mathrm{fou},j} & \ldots \end{array} & f_{\mathrm{fou},n} \end{array}\right].$$

Therefore the vector **u**_obj_ of displacement of all *n* points of the elevated tank can be expressed as

(12) $$u_{\mathrm{obj}}=f_{\mathrm{obj}}u_{\mathrm{ext}}$$

and the vector **u**_fou_ of displacement of all *n* jack supports can be expressed as

(13) $$u_{\mathrm{fou}}=f_{\mathrm{fou}}u_{\mathrm{ext}}.$$

The displacement vector **u**_o-f_ for the elevated tank against the jack support was equal to

(14) $$u_{o-f}=u_{\mathrm{obj}}-u_{\mathrm{fou}}$$

and using the Equations (6) and (14), it can be expressed as

(15) $$u_{o-f}=\left(f_{\mathrm{obj}}-f_{\mathrm{fou}}\right)k^{-1}Q.$$

If the elements *f*_fou,*i*,*j*_ of the matrix **f**_fou_ tend to zero, which corresponds to the rigid jack supports, then the displacements **u**_obj_ can be expressed as

(16) $$u_{\mathrm{obj}}=f_{\mathrm{obj}}k^{-1}Q.$$

The work *W_j_* done by the active support (*i* = *j*), in which there is the force *Q_j_*, resulted from the work of this force acting upon the forced piston extension *u*_ext,*j*_ and deformation of the whole structure, was equal to

(17) $$W_{\mathrm{jack},j}=Q_{i}u_{\mathrm{ext},j}+\frac{1}{2}k_{j,j}u_{\mathrm{ext},j}^{2}.$$

The force value *Q_i_* in the *i*-th jack resulted from the value *Q_g_*_,*i*_ and the history of forced piston extensions *u*_ext_. Assuming that the piston extension was forced in the jacks denoted by letters from *r* to *s,* then

(18) $$Q_{i}=Q_{g,i}+\overset{s}{\underset{j=r}{\sum }}k_{i,j}u_{\mathrm{ext},j}.$$

Based on the Equations (17) and (18), the relationship was derived for work *W* done by all *n* jacks when the forced extension in each of them was equal *u*_ext,*j*_ = *u*_ext_

(19) $$W=u_{\mathrm{ext}}\overset{n}{\underset{j=1}{\sum }}Q_{g,j}+u_{\mathrm{ext}}^{2}\left(\frac{1}{2}\overset{n}{\underset{j=1}{\sum }}k_{j,j}+\overset{n}{\underset{i=2}{\sum }}\overset{i-1}{\underset{j=1}{\sum }}k_{i,j}\right).$$

The expression (19) is a sum of two members. The first member, where the displacement *u*_ext_ occurred, expresses a change in potential energy of the construction as the rigid body. The second member at the symbol (*u*_ext_)^2^ defines a change in potential energy of deformed jacks, the elevated part and the supports.

## 3. Purpose and Plan of Tests

**Figure 4:**
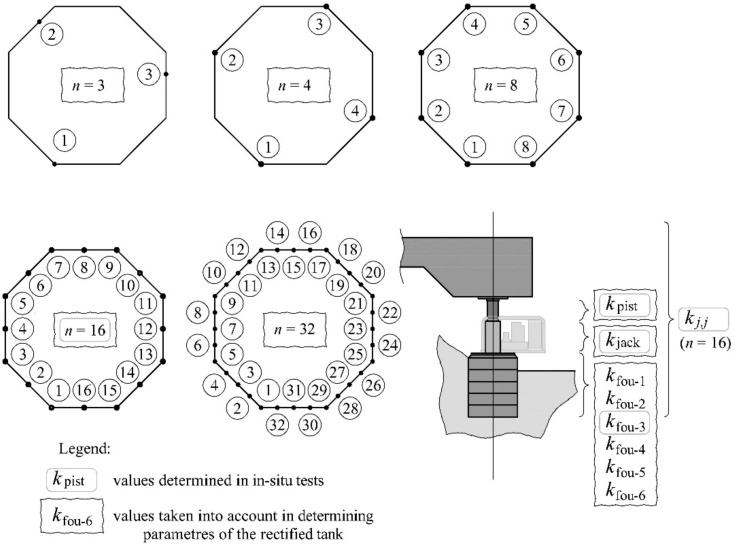
Values determined during the in-situ tests (*k*_pist_, *k*_jack_, *k*_fou-3_) and the model variables (*n*, *k*_fou_), for which the parameters **k**, **f**_obj_ and **f**_fou_ of the rectified tank were calculated (*k*_fou-1_ < *k*_fou-2_ < *k*_fou-3_ < *k*_fou-4_ < *k*_fou-5_ < *k*_fou-6_).

The purpose of the tests was to determine parameters **k**, **f**_obj_ and **f**_fou_ of the rectified tank, including real stiffness of elements of the tank supports. Therefore, in-situ testing of the tank placed on *n* = 16 jacks was performed before and after the rectification. These tests were used to determine stiffness for the support elements *k*_jack_, *k*_pist_, *k*_fou_, and the stiffness *k_j_*_,*j*_ (Figure 4). Then, the searched parameters were determined for these stiffness values and the taken model for the rectified tank.

Moreover, the aim of these tests was to analyse the effect of the model variables, which include the *n* number of the jacks and the jack support stiffness *k*_fou_, on the analysed parameters **k**, **f**_obj_ and **f**_fou_. The analyses were conducted for the *n* number of jacks equal to 3, 4, 8, 16, 32 (Figure 4) and six different stiffness values for the jack support *k*_fou-1_, *k*_fou-2_, *k*_fou-3_, *k*_fou-4_, *k*_fou-5_ and *k*_fou-6_. The stiffness *k*_fou-3_ was determined from the tests, whereas other values were freely chosen provided that *k*_fou-1_ < *k*_fou-2_ < *k*_fou-3_ < *k*_fou-4_ < *k*_fou-5_ < *k*_fou-6_. The aim of these analyses also consisted in expressing the effect of the model variables on the work done by the jacks during the elevation of the tank.

**Figure 5:**
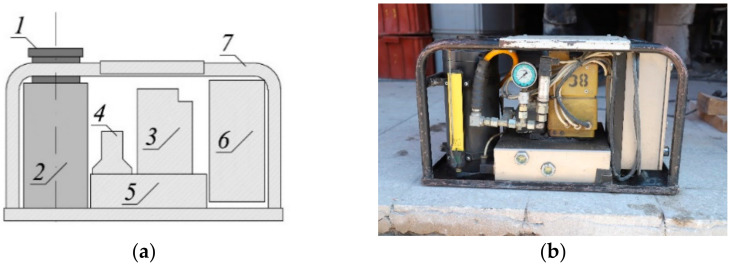
Hydraulic jack: (**a**) scheme; (**b**) view: 1—piston, 2—cylinder, 3—oil pump, 4—solenoid valves, 5—oil tank, 6*—*control box, 7—frame.

The hydraulic piston jacks were used for the in-situ tests and the rectification process (Figure 5). The jack was composed of the elements that transmitted loads: piston (1), cylinder (2) and other items, such as: oil pump (3), solenoid valves (4), oil tank (5), control box (6) and frame (7). The jacks rested on the jack supports which were made of stacks of concrete blocks pressed under the foundation slab. The stacks were pressed when the tank was filled with water, whereas the in-situ tests and the rectification were conducted after pumping out water from the tank.

### 3.1. In-Situ Testing

**Figure 6:**
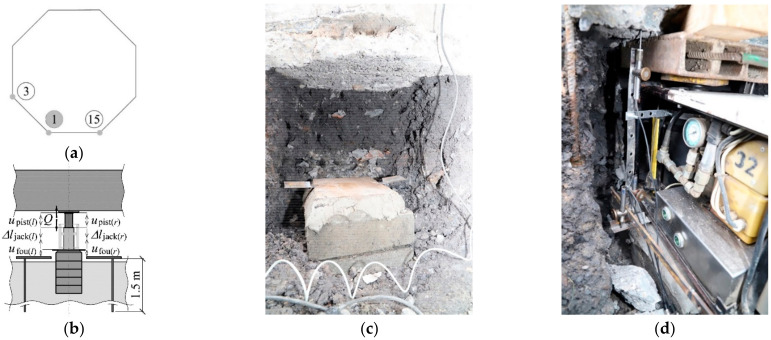
In-situ testing of stiffness *k*_jack_, *k*_pist_ and *k*_fou_: (**a**) position of the installed passive jack (1) and active jacks (3, 15); (**b**) plan of measurements (passive jack); (**c**) jack support in the form of a stack of concrete blocks pressed under the foundation slab; (**d**) jack placed on the jack support and prepared for tests.

**Figure 7:**
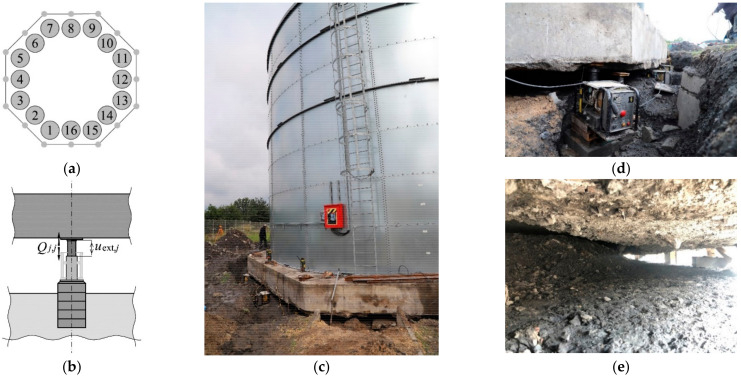
In-situ testing of stiffness *k_j_*_,*j*_ (*j* = 1, …, 16), (**a**) positions of the installed jacks and points of measuring the values *u*_ext*,j*_ and *Q_j,j_*; (**b**) plan of measurements; (**c**) tank placed on *n* = 16 jacks; (**d**) jack prepared for the tests, (**e**) space formed under the foundation slab after the rectification.

The in-situ tests were planned to be performed in two stages. The following stiffness values for the support elements were determined prior to the rectification: *k*_jack_, *k*_pist_ and *k*_fou_. For this purpose, the jacks were placed on previously prepared jack supports at points 1, 3 and 15 (Figure 6a) under the slab. The jack 1 was a passive one, while the piston extension in the jacks 3 and 15 was cyclically increasing or decreasing. Thus, a change in the force generated by the passive jack (1) was forced within the range from *Q*_min_ to *Q*_max_. The force *Q* and associated displacements *u*_fou_, *u*_pist_ as well as a change in the length ∆*l*_jack_ of the jack cylinder were measured during the tests. The force was measured by recording oil pressure in the jack. The displacements and a change in the cylinder length were measured using two linear variable differential transducers (LVDT) with an accuracy of 0.001 mm by measuring *u*_fou(*r*)_, *u*_fou*(l*)_ and *u*_pist(*r*)_, *u*_pist(*l*)_, and also ∆*l*_jack(*r*)_, ∆*l*_jack(*l*)_ (Figure 6b). Displacements *u*_fou(*r)*_, *u*_fou(*l*)_ of the jack supports against the ground were measured by measuring the displacement of the top surface area of the jack support (Figure 6c) against the bars driven into the ground to a depth of 1.5 m. Other measurements were taken for relative displacements of adequate points of the structure elements (Figure 6d). The places where the measurements were made and the measured values are shown in Figure 7a,b. The tank, while determining the parameters *k_i,j_*, is shown in Figure 7c,d.

The stiffness *k_j,j_* of the structure at the points *j* (*j* = 1, …, 16) defined by the expression (1) were determined after the rectification. Consequently, the piston extension *u*_ext,*j*_ in the *j*-th jack was forced for each support and a change in the force *Q_j,j_* produced by a given jack was recorded.

### 3.2. In-Situ Testing

Parameters of the tank placed on the jacks were determined from numerical calculations performed for the model of the structure. These parameters included the stiffness matrix **k**, the matrix **f**_obj_ of displacement forms of the elevated tank and the matrix **f**_fou_ of displacement forms of jack supports. The stiffness values *k*_fou_, *k*_jack_ and *k*_pist_ determined from the in-situ tests, were taken for this model. The elements *k_j_*_,*j*_ of the matrix **k** were compared with the values *k_j_*_,*j*_ determined during the in-situ tests.

**Figure 8:**
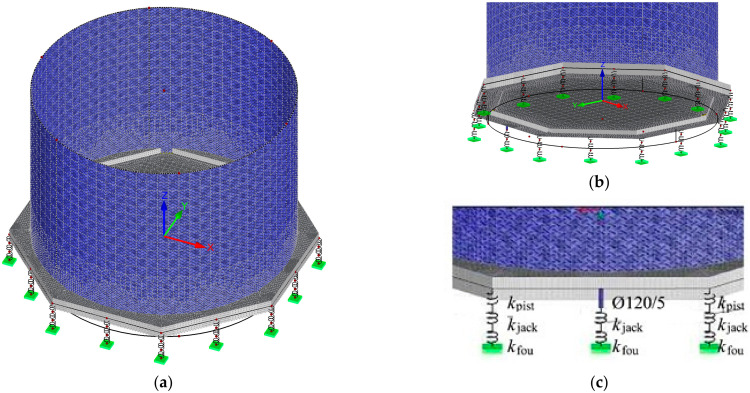
Tank model: (**a**) general view, (**b**) foundation slab; (**c**) passive supports and active support (inside).

The analysed model (Figure 8a) was composed of the reinforced concrete foundation slab, the steel tank, the jacks and the jack support. A slab of variable thickness in accordance with the survey (Figure 1 and Figure 2) having elasticity modulus *E*_c_ = 28.3 GPa, shear modulus *G*_c_ = 11.79 GPa, the Poisson’s ratio *ν* = 0.2 and weight density *γ*_c_ = 25 kN/m^3^, was used as the model of reinforced concrete foundation slab (Figure 8b). A shell of 3 mm in thickness, made of the material having elasticity modulus *E*_s_ = 200 GPa, shear modulus *G*_s_ = 76 GPa, the Poisson’s ratio *ν* = 0.3 and weight density *γ*_s_ = 78.5 kN/m^3^, was used as the model of steel tank. The hinged connection was used as the connection between the foundation slab and the tank.

The passive jacks were modelled with two stiffness values *k*_pist_ and *k*_jack_, which were connected in series and modelled stiffness of the piston and the jack cylinder (Figure 8c). These stiffness values were taken on the basis of the in-situ tests. The model of the active jack was also composed of two elements: one element with stiffness *k*_jack_ which modelled piston cylinder and steel pipe having a diameter of 120 mm, wall thickness of 5 mm and length of 300 mm, which was used as the piston model. The support with stiffness *k*_fou_ was used as the model of the jack support.

## 4. Results from In-Situ Tests

The in-situ tests were conducted after preparing supports for the jacks; that is, after pressing stacks of concrete blocks into ground at each of *n* = 16 points and removing water from the tank. The tank during the tests was placed on the supports which consisted of the jacks placed on their supports.

### 4.1. Stiffness of Support Elements

**Figure 9:**
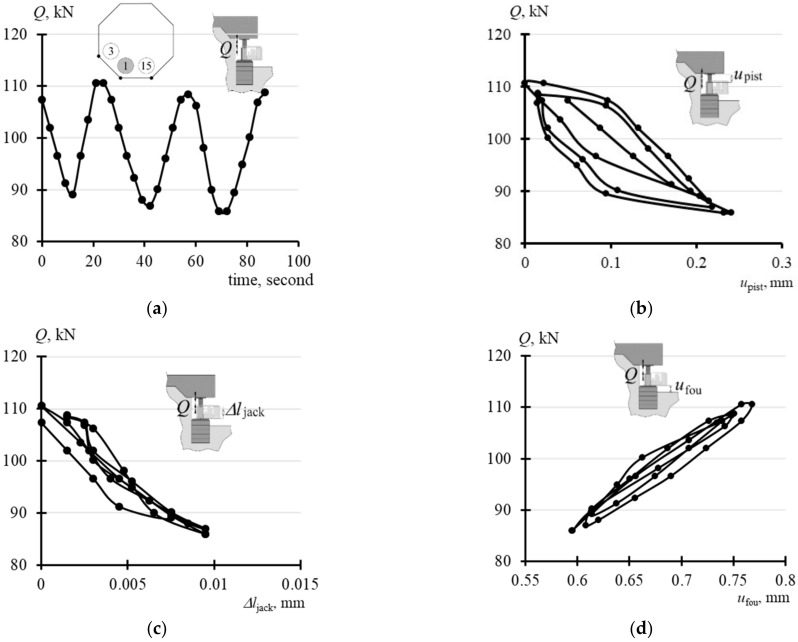
In-situ measurement results at the position of the passive jack: (**a**) *Q*—time; (**b**) *Q*—*u*_pist_; (**c**) *Q*—∆*l*_jack_; (**d**) *Q*—*u*_fou_; 1, 3, 15 – jack number.

Changes in the force *Q* in the passive jack (1*-* Figure 6a) were caused by the forced extension of pistons at the adjacent supports (3 and 15). Figure 9a presents three cycles of changes of the force *Q* within a range from *Q*_min_ to *Q*_max_. Changes in the force *Q* in the passive jack resulted in: changes in free extension of the piston *u*_pist_ (Figure 9b), changes in the length of the jack cylinder ∆*l*_jack_ (Figure 9c), and displacements of the jack support *u*_fou_ (Figure 9d). The maximum and minimum values *u*_pist_, ∆*l*_jack_ and *u*_fou_ corresponding to the values *Q*_max_ and *Q*_min_ were determined for the loops shown in Figure 9b–d and compared in Table A1 (Appendix A). These values were used to determine stiffness *k*_pist_, *k*_jack_ and *k*_fou_ for each loop as changes in the force values (*Q*_max_–*Q*_min_) divided by the relevant change (*u*_pist,max_–*u*_pist,min_), (∆*l*_jack,max_–∆*l*_jack,min_) and (*u*_fou,max_–*u*_fou,min_). Moreover, Table A1 presents the adequate mean stiffness values for three loops which were as follows: *k*_pist_ = 110 MN/m, *k*_jack_ = 2579 MN/m and *k*_fou_ = 150 MN/m.

### 4.2. Stiffness of Rectified Tank

**Figure 10:**
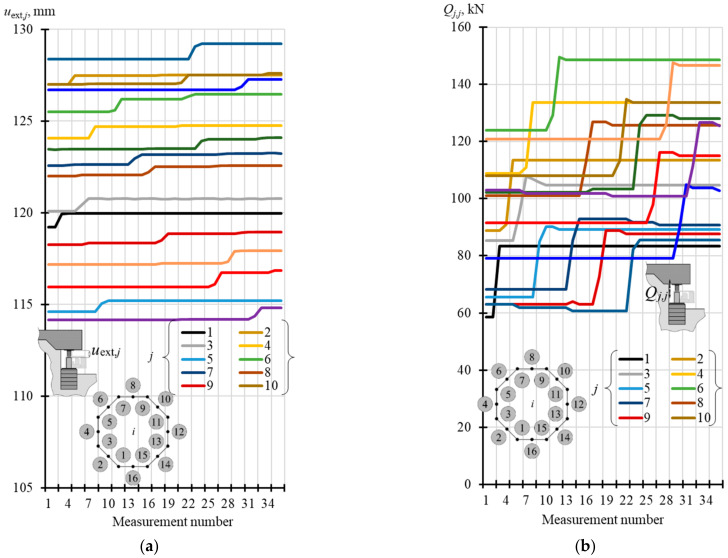
Results from in-situ tests for active supports (**a**) recorded forced piston extensions *u*_ext,*j*_; (**b**) recorded changes in forces *Q_j,j_* in the jacks.

The extension *u*_ext,*i*_ of the pistons was forced by a change in oil pressure in the consecutive jacks. Hence, for each *n* = 16 jack placed under the tank, the forced extension of piston was equal to *u*_ext,*j*_ (*j* = 1, …, 16) as presented in Figure 10a. The corresponding changes in the force *Q_j,j_* (*j* = 1, …, 16) of the jacks are shown in Figure 10b. The values of piston extension before increasing oil pressure in the *j*-th jack were denoted as *u*_ext,*j*,I_ and the extension after increasing oil pressure was denoted as *u*_ext,*j*,II_. The corresponding force values of the *j*-th jack were denoted as *Q_j_*_,I_ and *Q_j_*_,II_.

**Figure 11:**
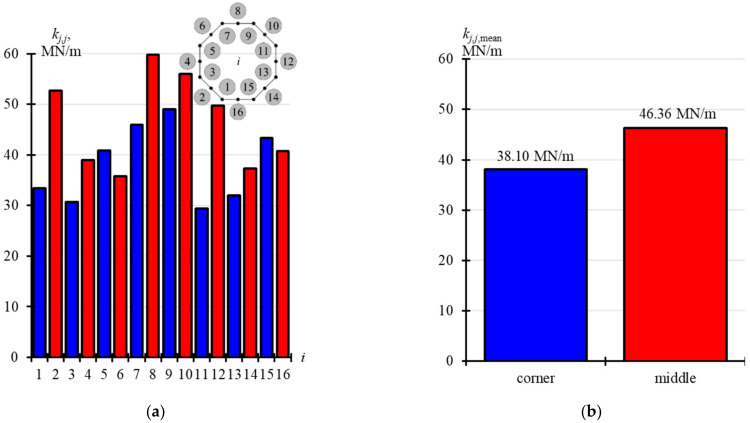
Results from in-situ tests for stiffness *k_j,j_*: (**a**) stiffness determined for *n* = 16 supports; (**b**) mean stiffness values corresponding to points in the slab corners (corner) and at the mid-point of the slab side (middle).

The values of *Q_j_*_,I_, *Q_j_*_,II_, *u*_ext,*j*,I_ and *u*_ext,*j*,II_ are compared in two tables. Table A2 (Appendix A) presents the values corresponding to the jacks installed in the corners of the foundation slab (*j* = 1, 3, 5, 7, 9, 11, 13, 15), while the values corresponding to the jacks installed at the midpoint of the foundation side (*j* = 2, 4, 6, 8, 10, 12, 14, 16) are summarised in Table A3 (Appendix A). Stiffness values *k_j,j_* in these tables were determined as relevant values (*Q_j,j_ = Q_j_*_,II_ − *Q_j_*_,I_) divided by (*u*_ext,*j*_ = *u*_ext,*j,*II_ − *u*_ext,*j,*I_). The determined values *k_j,j_* are displayed in Figure 11a. Figure 11b shows in blue mean stiffness values *k_j,j_* corresponding to the jacks installed in the corners (*k_j_*_,*j*,mean_ = 38.10 MN/m), and in red mean stiffness values *k_j,j_* corresponding to the jacks installed at the midpoint of the side (*k_j_*_,*j*,mean_ = 46.36 MN/m).

### 4.3. Analysis of the Model

The calculations were made for this model taking into account stiffness of the support elements determined from the in-situ tests which were as follows: *k*_pist_ = 110 MN/m, *k*_jack_ = 2579 MN/m and *k*_fou_ = 150 MN/m. They were used to verify correctness of the used numerical model of the rectified tank.

**Figure 12:**
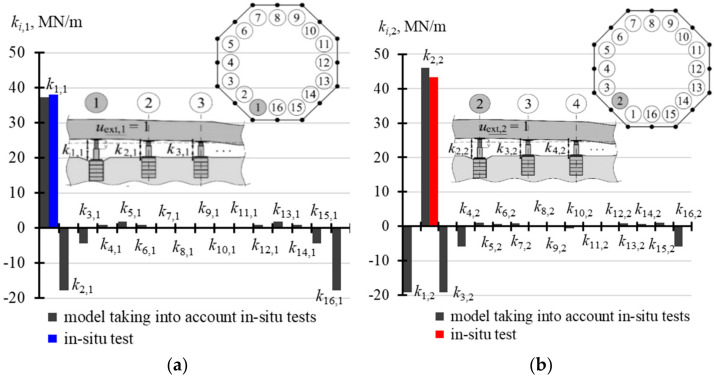
Values of elements in the matrix **k**: (**a**) elements *k_i_*_,1_; (**b**) elements *k_i_*_,2_.

**Figure 13:**
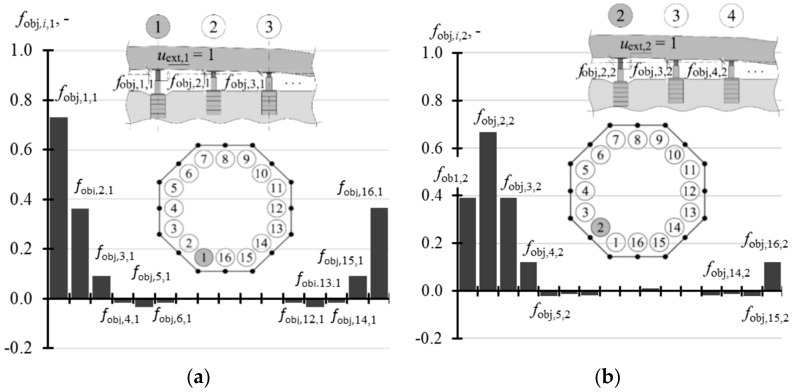
Values of elements in the matrix **f**_obj_: (**a**) elements *f*_obj,*i*,1_; (**b**) elements *f*_obj,*i*,2_.

**Figure 14:**
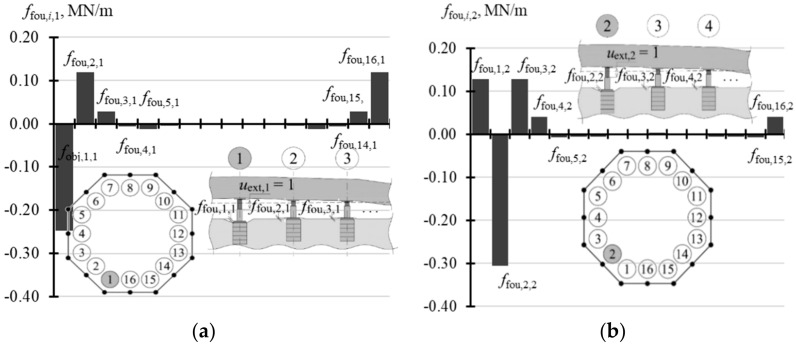
Values of elements in the matrix **f**_fou_: (**a**) elements *f*_fou,*i*,1_; (**b**) elements *f*_fou,*i*,2_.

The calculated results were the parameters **k**, **f**_obj_ and **f**_fou_ which formed the matrices, each in size of 16 × 16. Each *j*-th column was a vector corresponding to the effects produced by forced piston extension in the *j*-th jack by the unit value. The first column described the effects caused by the piston extension in the jack installed at the point *j* = 1 by *u*_ext,1_ = 1, the second column specified the effects produced by the piston extension in the jack installed at the point *j* = 2 by the unit value *u*_ext,2_ = 1, etc. Due to the structure symmetry, the column 3 related to the effects produced by the piston extension in the jack installed at the point *j* = 3 had the same values as the first column. However, the values in the column 3 of the matrix were finally shifted by two positions with reference to the column 1 in such a way that for the matrix **k** we obtain *k*_1,1_ = *k*_3,3_ = … = *k*_15,15_ and *k*_2,2_ = *k*_4,4_ = … = *k*_16,16_. Thus, values of the elements from two columns defined each of the matrices **k**, **f**_obj_ and **f**_fou_. For the matrix **k** these values were *k_i_*_,1_ and *k_i_*_,2_ (*i* = 1, …, 16), for the matrix **f**_obj_ they were vectors **f**_obj,1_ and **f**_obj,2_ having values equal to *f*_obj,*i*,1_ and *f*_obj,*i*,2_ (*i* = 1, …, 16), and for the matrix **f**_fou_ they were vectors **f**_fou,**1**_ and **f**_fou,2_ of values *f*_fou,*i*,1_ and *f*_fou,*i*,2_ (*i* = 1, …, 16). These values are summrised in Table A4 (Appendix A). While analysing the values of the stiffness matrix shown in this table, it should be emphasized that the experimentally determined value *k_j,j_* for the model corresponding to the jack installed in the corner was equal to 37.260 MN/m, and the mean value *k_j_*_,*j*_ for *j* = 1, 3, …, 15, which was determined from the in-situ tests, was equal to 38.10 MN/m. Moreover, the calculated value *k_j,j_* related to the jack installed at the mid-point of the foundation slab was 45.960 MN/m, and the relevant mean value *k_j,j_* determined from the in-situ tests for *j* = 2, 4, …, 16 was equal to 43.36 MN/m. Differences between these values which were experimentally obtained and determined for the model were equal to 6%, which was considered as the satisfactory conformity. These values presented in Table A4 are illustrated in Figure 12, Figure 13 and Figure 14.

The analysis of Figure 12 indicates that stiffness *k*_2,2_ was greater than stiffness *k*_1,1_. Moreover, the forced displacement *u*_ext,*j*_ in the *j*-th active jack caused an increase in the force. Simultaneously, a drop in the force was observed for the adjacent passive jacks. While the force in the jack 1 was exerted, the force dropped in four adjacent jacks (elements *k*_2,1_, *k*_3,1_ and *k*_16,1_, *k*_15,1_ of negative values—Figure 12a). Accordingly, when force in the jack 2 was exerted, the force dropped also in four adjacent jacks (elements *k*_1,2_, *k*_16,2_ and *k*_3,2_, *k*_4,2_ of negative values—Figure 12b). A minor increase or drop in the force values was observed in other passive jacks.

Considering the value *f*_obj,*i*,*j*_, it could be concluded that at the point, at which the piston extension (*i* = *j*) was forced, ca.70% of this extension (*f*_obj,1,1_ = 0.730, *f*_obj,2,2_ = 0.667) was transferred to the motion towards the top of the tank slab at that point. Moreover, the upward displacements were found for four adjacent points with reference to the points, at which the active jacks were operated (Figure 13a,b). Other points were shifted downwards or their displacement values were close to zero.

For the value *f*_fou,*i*,*j*_, the support for the jack, for which the extension *u*_ext,*j*_ = 1 of the piston (*i* = *j*) was forced, was observed to be displaced downwards. This displacement was equal to *f*_fou,1,1_ = 0.287 for the jacks installed in the corners, and *f*_fou,1,1_ = 0.306 for the jacks installed at the mid-point of the side, which contributed to 28.7–30.6% of the forced value *u*_ext_. The supports for four adjacent jacks were displaced upwards, while others did not move or were slightly displaced downwards (Figure 14a,b).

## 5. Analysis of the Structure Variables on Parameters of the Rectified Tank

The analysis further focused on the effect of variables *n* and *k*_fou_ of the model (*n* = 3, 4, 8, 16 and 32, *k*_fou_ = 50 MN/m, 100 MN/m, 150 MN/m, 200 MN/m, 250 MN/m and *k*_fou_—rigid) on the matrices **k**, **f**_obj_ and **f**_fou_ of the structure, which was the tank on the jacks that were placed on the jack supports.

### 5.1. Stiffness Matrix

The stiffness matrix **k** for the structure was a rectangular array of elements *k_i,j_* of the size *n* × *n*, where *n* is the number of the jacks, on which the tank was placed. For *n* = 3, all elements *k_i,j_* were equal to zero, which means that the forced extension *u*_ext,*j*_ in any *j*-th jack did not cause a change in the force *Q_i,j_* in any support. In other cases (*n* > 3), the diagonal elements *k_j,j_* (*i* = *j*) were always different than zero and took positive values, which means that the forced extension *u*_ext,*j*_ of the jack piston at the *j*-th support caused an increase in the force *Q_j,j_* (*i* = *j*) in the *j*-th support. Other matrix elements could take negative, positive or zero values. For the element *k_i,j_* lower than zero, the extension *u*_ext,*j*_ caused a drop in the force in the *i*-th support.

When *n* = 4, the values of three elements of the matrix found in the first column: *k*_1,1_, *k*_2,1_ and *k*_3,1_ were sufficient for the explicit description of the matrix **k**. The elements for the analysed stiffness *k*_fou_ of the supports are presented in Table A5 (Appendix A). Other elements from the first column, due to the symmetric arrangement of all jacks in the corners, could be determined by taking into account the matrix symmetry. Thus, the element *k*_4,1_ was equal to *k*_2,1_. Additionally, all diagonal elements were equal to each other. Thus, other columns could be also determined as the matrix was symmetric.

A similar situation occurred when *n* = 8. Considering the position of the jacks in the slab corners, which was a regular hexagon, first five elements of the first column: *k*_1,1_, *k*_2,1_, *k*_3,1_, *k*_4,1_, *k*_5,1_ were sufficient to define the whole matrix. Other elements of the matrix were determined as all diagonal elements were equal and the matrix was symmetrical. The elements describing the matrix **k**, when *n* = 8, for the analysed values *k*_fou_ are compared in Table A5 (Appendix A).

For *n* = 16, the matrix **k** had two different vectors. The first element *k_i_*_,1_ (*i* = 1, …, 16) defines changes in the values of forces in the supports caused by the unitary forced piston extension in the slab corner, and the second element *k_i_*_,2_ defines the values of forces in the supports caused by the unitary forced piston extension at the midpoint of the slab side. Each of the 16-element columns was clearly described by nine elements. Other elements of the first and second columns, and other columns could be determined from properties of the symmetric matrix taking into account that *k*_1,1_ = *k*_3,3_ = … = *k*_15,15_ and *k*_2,2_ = *k*_4,4_ = … = *k*_16,16_. The elements describing the matrix **k**, when *n* = 16, for the analysed values *k*_fou_ are compared in Table A6 (Appendix A).

Three columns had to be determined to define the matrix **k** when *n* = 32. Each of these columns was clearly described by 17 elements. Data presented in Table A7 (Appendix A) were reduced to first nine values of the vectors *k_i_*_,1_, *k_i_*_,2_, *k_i_*_,3_. The absolute value of other elements was lower than 3.27 MN/m.

**Figure 15:**
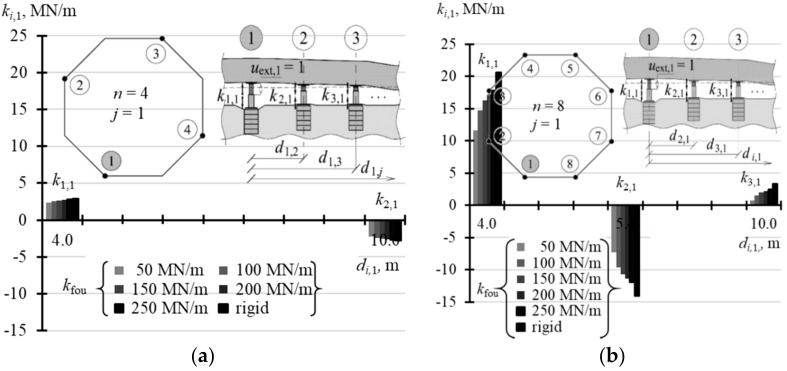
Selected elements of the stiffness matrix **k** for the structure for different stiffness values *k*_fou_ of the jack supports for: (**a**) *n* = 4; (**b**) *n* = 8.

**Figure 16:**
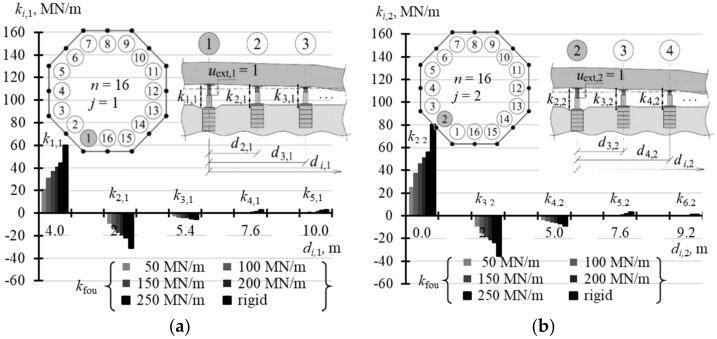
Selected elements of the stiffness matrix **k** for the structure for different stiffness values *k*_fou_ of the jack supports when *n* = 16: (**a**) elements *k_i,_*_1_; (**b**) elements *k_i,_*_2_.

**Figure 17:**
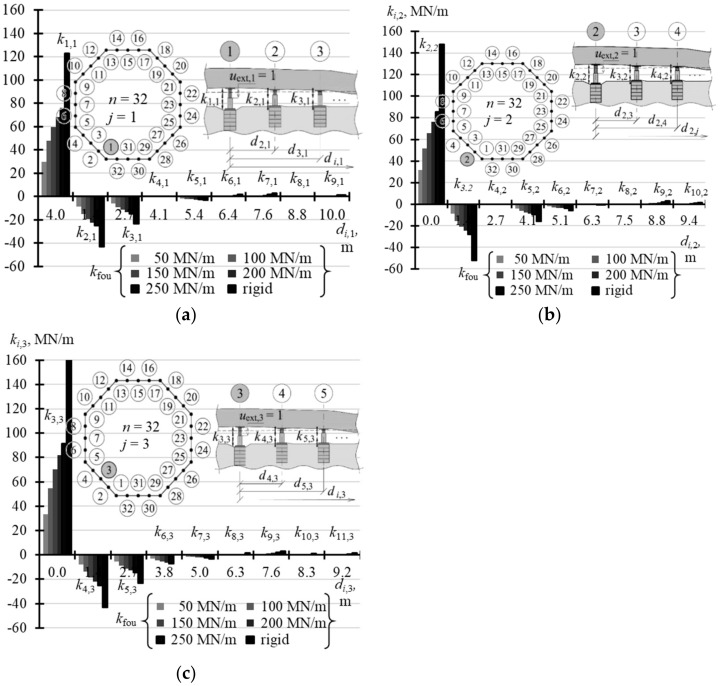
Selected elements of the stiffness matrix **k** for the structure for different stiffness values *k*_fou_ of the jack supports when: *n* = 32: (**a**) element*s k_i,_*_1_; (**b**) element*s k_i,_*_2_; (**c**) elements *k_i,_*_3_.

The selected elements of the matrix **k** for the analysed support stiffness *k*_fou_ are graphically presented in the following figures. Figure 15a illustrates the selected elements for *n* = 4, while Figure 15b shows the selected elements for *n* = 8. Figure 16a,b show selected elements *k_i_*_,1_ and *k_i_*_,2_ when *n* = 16. Figure 17a–c show selected elements of the columns *k_i_*_,1_, *k_i_*_,2_ and *k_i_*_,3_ for *n* = 32.

With an increasing *n* number of the jacks, on which the tank was placed, the values of diagonal elements *k_j,j_* were also increasing. At the same time, the structures of greater stiffness *k*_fou_ had higher values *k_j,j_*. For *n* = 4, the values *k*_1,1_ corresponding to stiffness *k*_fou_ = 50 MN/m were equal to 2.352 MN/m, and for the rigid support *k*_1,1_ were equal to 2.848 MN/m (Table A5 in Appendix A). Depending on the stiffness *k*_fou_, for *n* = 8, the values *k*_1,1_ ranged from 11.6 MN/m to 20.583 MN/m (Table A5); for *n* = 16 the values *k*_1,1_ ranged from 21.63 MN/m to 59.94 MN/m (Table A6 in Appendix A); and for *n* = 32 the values *k*_1,1_ ranged from 29.79 MN/m to 122.72 MN/m (Table A7 in Appendix A). The columns of the matrix corresponding to forced piston excitation on the jacks at the midpoint of the foundation side had higher values *k_j,j_*, for *n* = 16 and *n* = 32.

By analysing the *j*-th column of the matrix, it should be noted that the elements next to the diagonal element *k_j,j_* (*i* = *j*) always took negative values. For *n* = 4 and *n* = 8, two elements *k_i,i_*_-1_ and *k_i,i_*_+1_ adjacent to *k_j,j_* took negative values, for *n* = 16 six elements adjacent to *k_j,j_* took negative values, and for *n* = 32 eight elements adjacent to *k_i,i_* took negative values.

### 5.2. Matrix of Displacement Forms of Elevated Tank

**Figure 18:**
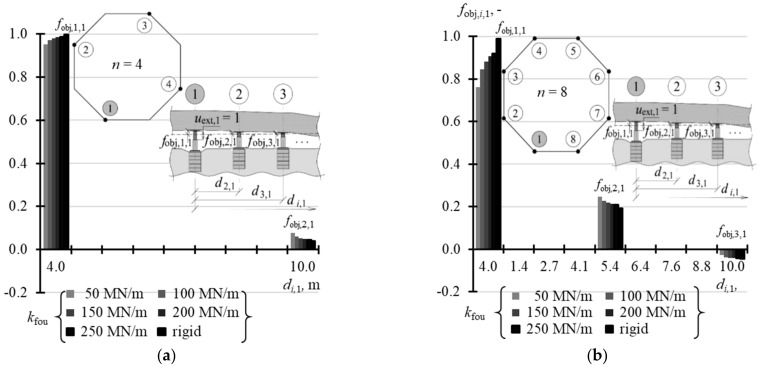
Elements of the matrix **f**_obj_ for different stiffness values *k*_fou_ of the jack support for: (**a**) *n* = 4; (**b**) *n* = 8.

**Figure 19:**
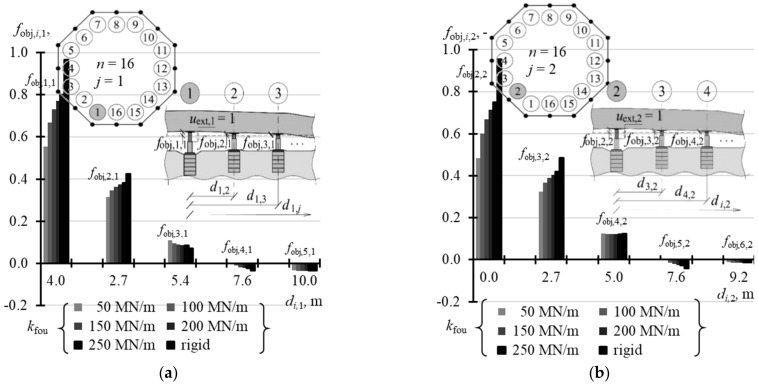
Elements of the matrix **f**_obj_ for different stiffness values *k*_fou_ of the jack support when *n* = 16: (**a**) elements *f*_obj*,i,*1_; (**b**) elements *f*_obj*,i,*2_.

**Figure 20:**
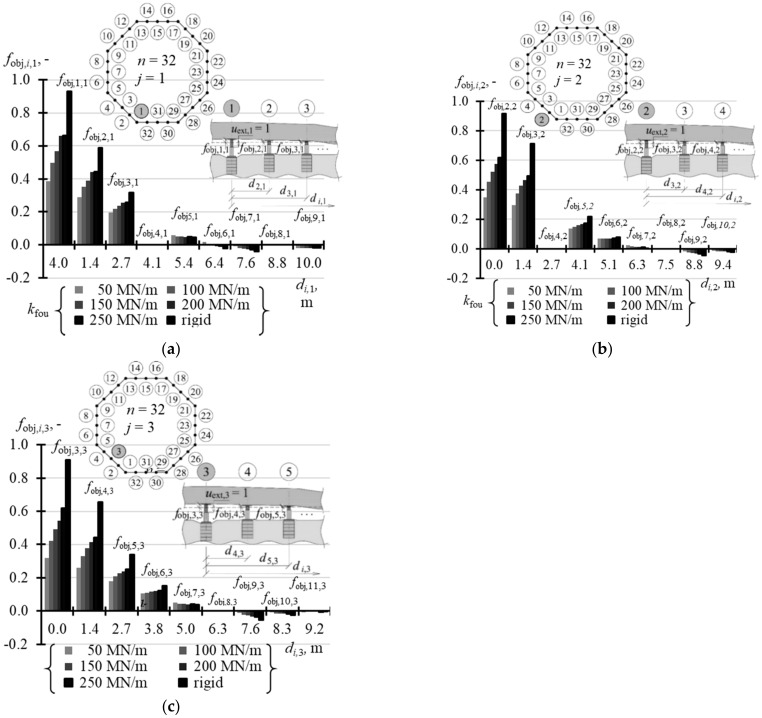
Elements of the matrix **f**_obj_ for different stiffness values *k*_fou_ of the jack support when *n* = 32: (**a**) elements *f*_obj*,i,*1_; (**b**) elements *f*_obj*,i,*2_; (**c**) element*s f*_obj*,i,*3_.

A structure of the matrix **f**_obj_ displacement forms of the elevated tank was similar to the structure of the stiffness matrix. This means that the matrix could be defined without the need to write the whole elements. Therefore, Table A8 (Appendix A) shows the selected values *f*_obj,*i*,1_ of the vector **f**_obj,1_ when *n* = 3, 4, and 8, Table A9 (Appendix A) contains the selected values of vectors **f**_obj,1_ and **f**_obj,2_ for *n* = 16, and Table A10 (Appendix A) presents the selected values of vectors **f**_obj,1_, **f**_obj,2_ and **f**_obj,3_ for *n* = 32. When *n* = 3, the elements *f*_obj,*j,j*_ (*i* = *j*) took the value 1, and the other elements *f*_obj,*i*,*j*_ (*i* ≠ *j*) took the value 0 regardless of the support stiffness *k*_fou_. In other cases, the values *f*_obj,*i*,*j*_ depended on stiffness *k*_fou_. The values *f*_obj,*j,j*_ (*i* = *j*) were higher for higher values *k*_fou_ and took lower values for a greater number *n* of the jacks. When *n* = 4, *f*_obj,*j,j*_ (*i* = *j*) took values from 0.952 to 0.998 (Figure 18a), and when *n* = 8 from 0.731 to 0.988 (Figure 18b). When *n* = 16, the values *f*_obj,*j,j*_ corresponding to the jacks installed in the corner took values from 0.555 to 0.966 (Figure 19a), and values from 0.484 to 0.954 in case of the jacks at the midpoint of the side (Figure 19b). When *n* = 32, the values *f*_obj,*j,j*_ (*i* = *j*) were within the range from 0.387 to 0.930 for the jacks in the corners (Figure 20a), from 0.350 to 0.916 for the jacks in ¼ of the side length (Figure 20b), and from 0.318 to 0.907 for the jacks in ½ of the side length (Figure 20c). 

### 5.3. Matrix of Displacement Forms of Jack Supports

The last test parameter of the tank was the matrix **f**_fou_ of displacement forms of jack supports, whose elements specified vertical displacements of the jack supports.

**Figure 21:**
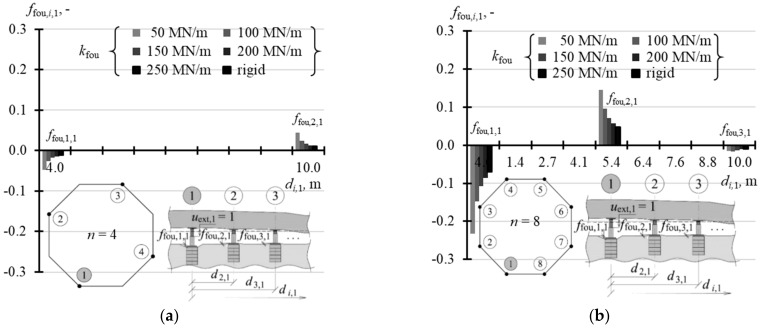
Elements of the matrix **f**_fou_ of support displacements for different stiffness values *k*_fou_ of the jack support: (**a**) *n* = 4; (**b**) *n* = 8.

**Figure 22:**
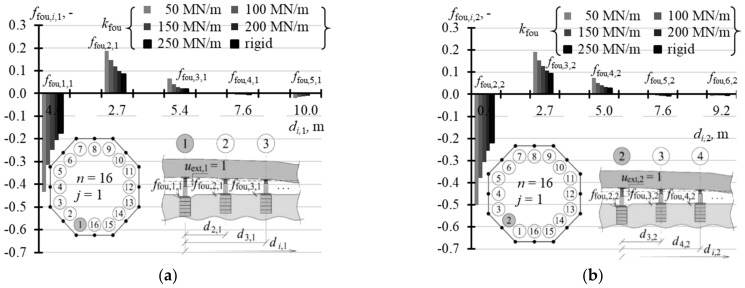
Elements of the matrix **f**_fou_ of support displacements for different stiffness values *k*_fou_ of the jack support when *n* = 16: (**a**) elements *f*_fou*,i,*1_; (**b**) elements *f*_fou*,i,*2_.

**Figure 23:**
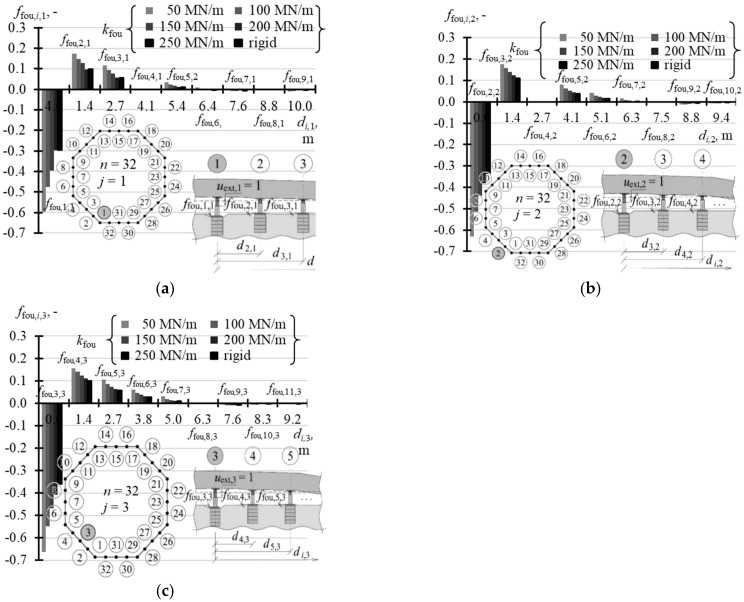
Elements of the matrix **f**_fou_ of support displacements for different stiffness values *k*_fou_ of the jack support when *n* = 32: (**a**) elements *f*_fou*,i,*1_; (**b**) elements *f*_fou*,i,*2_; (**c**) elements *f*_fou*,i,*3_.

For *n* = 3, the elements *f*_obj,*i*,*j*_ took the value 0 regardless of the support stiffness *k*_fou_ (Table A11 in Appendix A). In other cases, the values *f*_fou,*i*,*j*_ depended on stiffness *k*_fou_. The values *f*_fou,*j,j*_ (*i* = *j*) were higher for lower values *k_f_*_ou_ and took higher values for a greater number *n* of the jacks. When *n* = 4, *f*_fou,*j,j*_ (*i* = *i*) took values from −0.047 to −0.011 (Figure 21a), and when *n* = 8 from −0.232 to −0.070 (Figure 21b). The elements of vectors **f**_fou,1_ and **f_f_**_ou,2_ for *n* = 16 are compared in Table A12 (Appendix A). The values *f*_fou,*j,j*_ (*i* = *j*) corresponding to the jacks installed in the corner took values from −0.433 to −0.173 (Figure 22a), and from −0.484 to −0.219 for the jacks at the midpoint of the side (Figure 22b). When *n* = 32 (Table A13 in Appendix A), the values *f*_obj,*j,j*_ (*i* = *j*) were within the range from −0.596 to −0.296 for the jacks in the corners (Figure 23a), from −0.632 to −0.334 for the jacks in ¼ of the side length (Figure 23b), and from −0.663 to 0.359 for the jacks in ½ of the side length (Figure 23c). The values *f*_fou,*i*,*j*_ (*i* ≠ *j*) were higher or lower than zero. Higher absolute values *f*_obj,*i*,*j*_ (*i* ≠ *j*) were determined for lower values *k*_fou_, regardless of the number of jacks.

## 6. Analysis of Work Performed by Jacks

**Figure 24:**
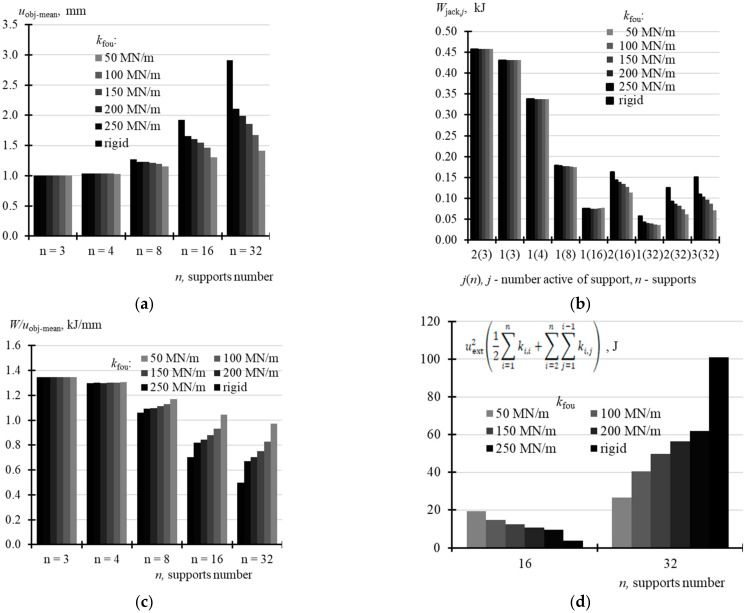
Analysed displacements of the tank and the work performed by the jacks: (**a**) mean displacements *u*_obj-mean_ derived from the Equation (20) at *u*_ext,*j*_ = 1 mm (*j* = 1, …, *n*); (**b**) work *W*_jack,*j*_ performed by the active jack; (**c**) work *W* obtained from (19) divided by mean displacements *u*_obj-mean_ from (20); (**d**) elastic energy of the deformed slab after forced displacements *u*_ext,*j*_ = 1 mm (*j* = 1, …, *n*) of pistons in all the jacks.

The further analysis focused on the forced piston extension *u*_ext,*j*_ by 1 mm, which was the same for all the jacks. For *n* = 3, the mean displacement of the structure *u*_obj-mean_ determined from the relationship

(20) $$u_{\mathrm{obj}-\mathrm{mean}}=\frac{1}{n}\overset{n}{\underset{i=1}{\sum }}\overset{n}{\underset{j=1}{\sum }}f_{\mathrm{obj},i,j}u_{\mathrm{ext},j}$$

was equal to 1 mm. In other cases, *u*_obj-mean_ determined from the relations (20) depended on both the *n* number of the jacks and the support stiffness *k*_fou_, and *u*_obj-mean_ was increasing as stiffness *k*_fou_ and the jack number *n* were increasing (Table A14 in Appendix A). When *n* = 4, then *u*_obj-mean_ ranged from 1.031 mm to 1.038 mm; when *n* = 8, *u*_obj-mean_ took values from 1.151 mm to 1.272 mm; when *n* = 16, *u*_obj-mean_ ranged from 1.307 mm to 1.923 mm; and when *n* = 32, *u*_obj,mean_ took values from 1.414 mm to 2.911 mm (Figure 24a). Assuming that the force *Q_g,i_*, the values of which for the analysed variables are shown in Table A15 (Appendix A), was acting on each active support prior to the forced extension *u*_ext_ of the pistons, then the work *W*_jack,*j*_ performed by the *j*-th active jack during the piston extension by *u*_ext_ = 1 mm depended on the number *n* of supports. Moreover, for *n* = 16 and 32, work *W*_jack,*j*_ depended also on the support stiffness *k*_fou_ (Figure 24b). Higher values *W*_jack,*j*_ were obtained for higher values *k*_fou_. For the jacks installed in the corner, the work took the following values: 0.458 kJ at *n* = 3, 0.338 kJ at *n* = 4, from 0.176 kJ to 0.179 kJ at *n* = 8, from 0.075 kJ to 0.076 kJ at *n* = 16, and from 0.037 kJ to 0.057 kJ at *n* = 32.

**Figure 25:**
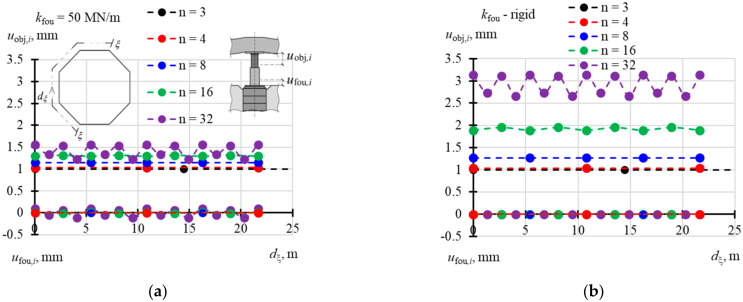
Displacements of the object at points *i* (*u*_obj,*i*_) and displacements of the support of the *i*-th jack (*u*_fou,*i*_) after forced extensions of all the jack pistons *u*_ext,*j*_ = 1 mm (*j* = 1, …, *n*): (**a**) *k*_fou_ = 50 MN/m; (**b**) rigid support for the jacks.

The total work *W* done by all the jacks during the piston extensions *u*_ext_ = 1 mm and determined from the relationship (19) was the same for *n* = 3, 4 and 8 jacks, did not depend on *k*_fou_ and was equal to1.347 kJ. For *n* = 16, this work depended on stiffness *k*_fou_ and was equal to 1.366 kJ at *k*_fou_ = 50 MN/m and 1.350 kJ at the rigid support (Table A14). For *n* = 32, this work was equal to 1.373 Mn/m at *k*_fou_ = 50 MN/m, and equal to 1.448 MN/m at the rigid support. These differences were a consequence of elastic deformation, to which the structure was subjected after the forced extension *u*_ext_ of all pistons. Displacements *u*_obj,*i*_ of the points on the slabs and displacements *u*_fou,*i*_ of the jack supports after forced extension of the pistons *u*_ext,*j*_ = 1 (*j* = 1, …, *n*) are illustrated in Figure 25a for *k*_fou_ = 50 MN/m, and in Figure 25b for the rigid support.

Table A14 shows the values of work described by the Equation (19) divided by the mean displacement *u*_obj-mean_ expressed as (20). This comparison indicates that lifting the building by 1 mm required the least energy when the number of supports was *n* = 32 and the jack support was rigid. Then, the result was 0.497 kJ/mm. Lifting the structure by 1 mm required the highest energy for *n* = 3 jacks. Then, the result was 1.367 kJ/mm. It can be concluded that installing a significant number of the jacks and providing the rigid jack support for them was the most favourable conditions during the rectification.

## 7. Conclusions

The parameters of the rectified tank placed on *n* hydraulic piston jacks installed under the tank were described using three matrices: stiffness matrix **k**, matrix **f**_obj_ of displacement forms of elevated tank, and matrix **f**_fou_ of displacement forms of jack supports. 

When *n* = 3, the structure was statically determinate. Hence, the matrices **k** and **f**_fou_ had entries equal to zero, and the matrix **f**_obj_ was the identity matrix. With an increasing number *n* of the jacks, on which the tank was placed, the values of diagonal elements *k_j,j_* were also increasing. At the same time, the structures of greater stiffness *k*_fou_ had higher values *k_j,j_*. For *n* = 4, the values *k_j,j_* corresponding to stiffness *k*_fou_ = 50 MN/m were equal to 2.352 MN/m, and 2.848 MN/m for the rigid support *k*_fou_. For *n* = 32, the values *k_j,j_* ranged from 29.79 MN/m to 122.72 MN/m. The values *k_i,j_* (*i* ≠ *j*) were lower for a greater number of the jacks and higher stiffness values *k*_fou_, which meant a bigger drop in forces in the active support at the same forced extension *u*_ext_.

The values *f*_obj,*j,j*_ (*i* = *j*) were higher for higher stiffness values *k*_fou_ and took lower values for a greater *n* number of the jacks. For *n* = 4, *f*_obj,*j,j*_ (*i* = *j*) took values from 0.952 to 0.998. For *n* = 32, the values *f*_obj,*j,j*_ (*i* = *j*) were within a range of 0.335–0.930.

The conducted energy analysis demonstrated that as the *n* number of jacks and stiffness *k*_fou_ were increasing, the amount of work required to provide the vertical displacement of the object was decreasing. For *n* = 3, the work required to elevate the object by 1 mm was 1.347 kJ. For *n* = 32 and the rigid support for the jacks, 0.497 kJ of energy was sufficient to elevate the object by 1 mm. Thus, it can be concluded that installing a significant number of the jacks and providing the rigid jack support for them was the most favourable condition during the rectification. On the other hand, increasing the number of jacks increases the investment cost. For this reason, the number of jacks used for the tank rectification was limited to 16.

## Data Availability

Not applicable.

## Appendix A

**Table A1:** Extreme values: *Q*, *u*_pist_, **∆***l*_jack_ and *u*_fou_ determined from subsequent load cycles of the passive support during the in-situ tests and stiffness of the support elements *k*_pist_, *k*_jack_, *k*_fou_ determined on their basis.

Loop	*Q*_max_	*u*_pist,min_	∆*l*_jack,min_	*u*_fou,min_	*k*_pist_	*k*_jack_	*k*_fou_
	*Q*_min_	*u*_pist,max_	∆*l*_jack,max_	*u*_fou,max_			
	[kN]	[mm]	[mm]	[mm]	[MN/m]	[MN/m]	[MN/m]
1	107.36	0.049	0.000	0.740	118	2433	145
	89.11	0.204	0.008	0.614			
2	110.58	0.000	0.000	0.758	108	2486	158
	86.96	0.218	0.010	0.608			
3	108.43	0.016	0.002	0.748	104	2818	147
	85.89	0.232	0.010	0.595			
mean values					110	2579	150

**Table A2:** Stiffness *k_j,j_* determined from in-situ tests according to (1) at points at the foundation corners.

*i* = *j*	1	3	5	7	9	11	13	15
*Q_j_*_,I_, kN	58.63	85.31	65.56	68.17	62.98	60.77	91.45	79.09
*Q_j_*_,II_, kN	83.32	105.71	90.25	92.86	88.75	85.47	116.14	103.78
*Q_j,j_* = *Q_j_*_,II_ − *Q_j_*_,I_, kN	24.69	20.40	24.69	24.69	25.77	24.69	24.69	24.69
*u*_ext,*j*,I_, mm	119.23	120.10	114.59	122.64	118.35	128.38	115.96	126.70
*u*_ext,*j*,II_, mm	119.97	120.76	115.20	123.18	118.87	129.22	116.74	127.27
*u*_ext,*j*_ = *u*_ext,*j*,II_ − *u*_ext,*j*,I_, mm	0.74	0.67	0.60	0.54	0.53	0.84	0.77	0.57
*k_j,j_* MN/m	33.44	30.66	40.87	45.98	49.05	29.43	31.99	43.28

**Table A3:** Stiffness *k_j,j_* determined from in-situ tests according to (1) at points at mid-points of the sides.

*i* = *j*	2	4	6	8	10	12	14	16
*Q_j_*_,I_, kN	88.84	108.84	123.84	101.05	107.95	103.32	120.77	100.80
*Q_j_*_,II_, kN	113.54	133.54	148.53	126.82	133.72	129.09	146.54	126.57
*Q_j,j_* = *Q_j_*_,II_ − *Q_j_*_,I_, kN	24.69	24.69	24.69	25.77	25.77	25.77	25.77	25.77
*u*_ext,*j*,I_, mm	127.00	124.06	125.49	122.07	127.04	123.50	117.24	114.19
*u*_ext,*j*,II_, mm	127.47	124.69	126.18	122.50	127.50	124.02	117.93	114.82
*u*_ext,*j*_ = *u*_ext,*j*,II_ − *u*_ext,*j*,I_, mm	0.47	0.63	0.69	0.43	0.46	0.52	0.69	0.63
*k_j,j_* MN/m	52.68	38.94	35.82	59.71	55.98	49.76	37.32	40.71

**Table A4:** Tank parameters calculated for *n* = 16 and *k*_jack_ = 2579 MN/m, *k*_pist_ = 110 MN/m and *k*_fou_ = 150 MN/m (elements, for which *i* = *j*, that is, *k_j,j_*, *f*_obj*,j,j*_ and *f*_obj*,j,j*_ are given in bold).

*i*	*k_i_*_,1_, MN/m	*k_i_*_,2_, MN/m	*f*_obj,*i*,1_, -	*f*_obj,*i*,2_, -	*f*_fou,*i*,1_, -	*f*_fou,*i*,2_, -
1	**37.260**	−19.142	**0.730**	0.390	**−0.248**	0.128
2	−17.853	**45.960**	0.363	**0.667**	0.119	**−0.306**
3	−4.407	−19.142	0.090	0.390	0.029	0.128
4	0.882	−5.943	−0.018	0.121	−0.006	0.040
5	1.765	1.019	−0.036	−0.021	−0.012	−0.007
6	0.794	0.702	−0.016	−0.014	−0.005	−0.005
7	0.238	0.896	−0.005	−0.018	−0.002	−0.006
8	−0.009	−0.281	0.000	0.006	0.000	0.002
9	−0.071	0.024	0.001	0.000	0.000	0.000
10	−0.009	−0.508	0.000	0.010	0.000	0.003
11	0.239	0.022	−0.005	0.000	−0.002	0.000
12	0.795	−0.28	−0.016	0.006	−0.005	0.002
13	1.764	0.896	−0.036	−0.018	−0.012	−0.006
14	0.884	0.702	−0.018	−0.014	−0.006	−0.005
15	−4.409	1.019	0.090	−0.021	0.029	−0.007
16	−17.858	−5.944	0.364	0.121	0.119	0.040

**Table A5:** Values *k_i,j_* of stiffness matrix **k** expressed in MN/m, for *n* = 3, *n* = 4 and *n* = 8 and *k*_fou_ = 50, 100, 150, 200, 250 MN/m and *k*_fou_*-*rigid (elements *k_j,j_* (*i* = *j*) are given in bold).

*n*	*k_i,j_*	*k*_fou_, MN/m					
		50	100	150	200	250	Rigid
3	k_1,1_	0	0	0	0	0	0
	k_1,2_	0	0	0	0	0	0
4	k_1,1_	**2.352**	**2.576**	**2.66**	**2.705**	**2.735**	**2.848**
	k_2,1_	−2.258	−2.478	−2.561	−2.605	−2.634	−2.746
	k_3,1_	2.164	2.381	2.463	2.506	2.534	2.644
8	k_1,1_	**11.6**	**14.747**	**16.257**	**17.145**	**17.778**	**20.583**
	k_2,1_	−7.303	−9.602	−10.726	−11.393	−11.87	−14.004
	k_3,1_	0.773	1.558	1.979	2.237	2.426	3.306
	k_4,1_	0.699	0.737	0.728	0.715	0.701	0.606
	k_5,1_	0.061	−0.134	−0.218	−0.263	−0.293	−0.402

**Table A6:** Values *k_i_*_,*j*_ of stiffness matrix **k** expressed in MN/m, for *n* = 16 and *k*_fou_ = 50, 100, 150, 200, 250 MN/m and *k*_fou_*-*rigid (elements *k_j,j_* (*i* = *j*) are given in bold).

*i*	*k*_fou_, MN/m											
	50		100		150		200		250		Rigid	
	*k_i_*_,1_	*k_i_*_,2_	*k_i_*_,1_	*k_i_*_,2_	*k_i_*_,1_	*k_i_*_,2_	*k_i_*_,1_	*k_i_*_,2_	*k_i_*_,1_	*k_i_*_,2_	*k_i_*_,1_	*k_i_*_,2_
1	**21.63**	−9.65	**31.48**	−15.45	**37.26**	−19.14	**41.08**	−21.69	**44.03**	−23.71	**59.94**	−35.47
2	−9.34	**25.07**	−14.62	**37.96**	−17.85	**45.96**	−20.02	**51.42**	−21.71	**55.72**	−30.99	**80.44**
3	−3.23	−9.65	−4.04	−15.45	−4.41	−19.14	−4.61	−21.69	−4.75	−23.71	−5.29	−35.47
4	−0.09	−3.71	0.49	−5.15	0.88	−5.94	1.16	−6.46	1.38	−6.85	2.63	−8.90
5	0.98	−0.06	1.50	0.57	1.77	1.02	1.93	1.34	2.04	1.60	2.57	3.17
6	0.58	0.36	0.73	0.59	0.79	0.70	0.83	0.76	0.86	0.80	0.98	0.93
7	0.30	0.62	0.27	0.81	0.24	0.90	0.22	0.95	0.20	0.99	0.09	1.21
8	0.01	0.01	−0.01	−0.16	−0.01	−0.28	0.00	−0.37	0.01	−0.44	0.08	−0.85
9	−0.03	0.02	−0.05	0.01	−0.07	0.02	−0.07	0.04	−0.07	0.05	−0.08	0.16

**Table A7:** Values *k_i_*_,*j*_ of stiffness matrix **k** expressed in MN/m, for *n* = 32 and *k*_fou_ = 50, 100, 150, 200, 250 MN/m and *k*_fou_*-*rigid (elements *k_j,j_* (*i* = *j*) are given in bold).

*i*	*k*_fou_, MN/m											
	50		100		150		200		250		Rigid	
	*k_i_*_,1_		*k_i_*_,1_		*k_i_*_,1_		*k_i_*_,1_		*k_i_*_,1_		*k_i_*_,1_	
	*k_i_*_,2_	*k_i_*_,3_	*k_i_*_,2_	*k_i_*_,3_	*k_i_*_,2_	*k_i_*_,3_	*k_i_*_,2_	*k_i_*_,3_	*k_i_*_,2_	*k_i_*_,3_	*k_i_*_,2_	*k_i_*_,3_
1	**29.79**		**47.57**		**59.61**		**68.32**		**75.46**		**122.72**	
	−8.74	−5.31	−15.32	−8.73	−20.08	−11.10	−23.66	−12.86	26.68	−14.33	−48.46	−24.84
2	−8.59		−14.84		−19.22		−22.44		−25.11		−43.04	
	**31.61**	−7.75	**51.71**	−13.96	**65.87**	−18.57	**76.39**	−22.11	**85.24**	−25.13	**148.23**	−47.75
3	−5.79		−9.29		−11.61		−13.25		−14.59		−23.18	
	−8.83	**33.15**	−15.81	**54.76**	−20.93	**70.16**	−24.80	**81.72**	−28.09	**91.50**	−52.03	**162.99**
4	0.00		0.00		0.00		0.00		0.00		0.00	
	0.00	−7.75	0.00	−13.96	0.00	−18.57	0.00	−22.11	0.00	−25.13	0.00	−47.754
5	−1.75		−2.21		−2.45		−2.60		−2.71		−3.31	
	−4.06	−5.31	−6.31	−8.73	−7.80	−11.10	−8.88	−12.86	−9.77	−14.33	−15.84	−24.843
6	−0.46		−0.19		0.04		0.22		0.37		1.46	
	−2.11	−3.11	−2.90	−4.67	−3.38	−5.68	−3.71	−6.40	−3.97	−7.00	−5.66	−11.073
7	0.24		0.75		1.11		1.37		1.58		2.98	
	−0.77	−1.48	−0.76	−1.87	−0.70	−2.06	−0.64	−2.17	−0.59	−2.26	−0.14	−2.729
8	0.00		0.00		0.00		0.00		0.00		0.00	
	0.00	0.00	0.00	0.00	0.00	0.00	0.00	0.00	0.00	0.00	0.00	1.46
9	0.58		0.84		0.96		1.04		1.09		1.35	
	0.54	0.36	1.06	0.94	1.41	1.37	1.66	1.69	1.86	1.96	3.27	0

**Table A8:** Values *f*_obj,*i*,*j*_, [-], of the matrix **f**_obj_ of object displacement forms for *n* = 3, 4, 8, *k*_fou_ = 50, 100, 150, 200, 250 MN/m and *k*_fou_-rigid (elements *f*_obj,*j,j*_ (*i* = *j*) are given in bold).

*n*	*f*_obj,*i,j*_	*k*_fou_, MN/m					
		50	100	150	200	250	Rigid
3	*f*_obj,1,1_	**1**	**1**	**1**	**1**	**1**	**1**
	*f*_obj,1,2_	0	0	0	0	0	0
4	*f*_obj,1,1_	**0.952**	**0.973**	**0.981**	**0.985**	**0.988**	**0.998**
	*f*_obj,2,1_	0.076	0.059	0.052	0.049	0.046	0.038
	*f*_obj,3,1_	−0.073	−0.056	−0.05	−0.047	−0.045	−0.036
8	*f*_obj,1,1_	**0.761**	**0.844**	**0.882**	**0.905**	**0.92**	**0.988**
	*f*_obj,2,1_	0.246	0.227	0.218	0.213	0.209	0.192
	*f*_obj,3,1_	−0.026	−0.037	−0.04	−0.042	−0.043	−0.045
	*f*_obj,4,1_	−0.024	−0.017	−0.015	−0.013	−0.012	−0.008
	*f*_obj,5,1_	−0.002	0.003	0.004	0.005	0.005	0.006

**Table A9:** Values *f*_obj,*i*,*j*_, [-] of the matrix **f**_obj_ of object displacement forms for *n* = 16, *k*_fou_ = 50, 100, 150, 200, 250 MN/m and *k*_fou_*-*rigid (elements *f*_obj,*i*,*i*_ (*i* = *j*) are given in bold).

*i*	*k*_fou_, MN/m											
	50		100		150		200		250		Rigid	
	*f*_obj,*i*,1_	*f*_obj,*i*,2_	*f*_obj,*i*,1_	*f*_obj,*i*,2_	*f*_obj,*i*,1_	*f*_obj,*i*,2_	*f*_obj,*i*,1_	*f*_obj,*i*,2_	*f*_obj,*i*,1_	*f*_obj,*i*,2_	*f*_obj,*i*,1_	*f*_obj,*i*,2_
1	**0.555**	0.325	**0.667**	0.366	**0.730**	0.390	**0.771**	0.405	**0.802**	0.418	**0.966**	0.486
2	0.315	**0.484**	0.346	**0.599**	0.363	**0.667**	0.374	**0.714**	0.382	**0.750**	0.424	**0.954**
3	0.109	0.325	0.096	0.366	0.090	0.390	0.086	0.405	0.084	0.418	0.072	0.486
4	0.003	0.125	−0.012	0.122	−0.018	0.121	−0.022	0.121	−0.024	0.121	−0.036	0.122
5	−0.033	0.002	−0.036	−0.014	−0.036	−0.021	−0.036	−0.025	−0.036	−0.028	−0.035	−0.043
6	−0.019	−0.012	−0.017	−0.014	−0.016	−0.014	−0.016	−0.014	−0.015	−0.014	−0.013	−0.013
7	−0.010	−0.021	−0.006	−0.019	−0.005	−0.018	−0.004	−0.018	−0.003	−0.017	−0.001	−0.017
8	0.000	0.000	0.000	0.004	0.000	0.006	0.000	0.007	0.000	0.008	−0.001	0.012
9	0.001	−0.001	0.001	0.000	0.001	0.000	0.001	−0.001	0.001	−0.001	0.001	−0.002

**Table A10:** Values *f*_obj,*i*,*j*_, [-] of the matrix **f**_obj_ of object displacement forms for *n* = 32, *k*_fou_ = 50, 100, 150, 200, 250 MN/m and *k*_fou_*-*rigid (elements *f*_obj,*i*,*i*_ (*i* = *j*) are given in bold).

*i*	*k*_fou_, MN/m											
	50		100		150		200		250		Rigid	
	*f*_obj,*i*,1_		*f*_obj,*i*,1_		*f*_obj,*i*,1_		*f*_obj,*i*,1_		*f*_obj,*i*,1_		*f*_obj,*i*,1_	
	*f*_obj,*i*,2_	*f*_obj,*i*,3_	*f*_obj,*i*,2_	*f*_obj,*i*,3_	*f*_obj,*i*,2_	*f*_obj,*i*,3_	*f*_obj,*i*,2_	*f*_obj,*i*,3_	*f*_obj,*i*,2_	*f*_obj,*i*,3_	*f*_obj,*i*,2_	*f*_obj,*i*,3_
1	**0.387**		**0.497**		**0.569**		**0.661**		**0.661**		**0.930**	
	0.294	0.179	0.363	0.207	0.409	0.226	0.442	0.240	0.470	0.252	0.664	0.654
2	0.290		0.352		0.391		0.442		0.442		0.589	
	**0.350**	0.261	**0.453**	0.331	**0.523**	0.378	**0.575**	0.413	**0.617**	0.443	**0.916**	0.654
3	0.195		0.220		0.236		0.257		0.257		0.317	
	0.297	**0.318**	0.374	**0.421**	0.426	**0.492**	0.464	**0.545**	0.495	**0.617**	0.712	**0.907**
4	0.000		0.000		0.000		0.000		0.000		0.000	
	0.000	0.261	0.000	0.331	0.000	0.378	0.000	0.413	0.000	0.443	0.000	0.654
5	0.059		0.052		0.050		0.048		0.048		0.045	
	0.137	0.179	0.149	0.207	0.159	0.226	0.166	0.240	0.172	0.252	0.217	0.340
6	0.016		0.005		−0.001		−0.007		−0.007		−0.020	
	0.071	0.105	0.069	0.111	0.069	0.116	0.069	0.120	0.070	0.123	0.078	0.152
7	−0.008		−0.018		−0.023		−0.028		−0.028		−0.041	
	0.026	0.050	0.018	0.044	0.014	0.042	0.012	0.041	0.010	0.040	0.002	0.037
8	0.000		0.000		0.000		0.000		0.000		0.000	
	0.000	0.000	0.000	0.000	0.000	0.000	0.000	0.000	0.000	0.000	0.000	0.000
9	−0.020		−0.020		−0.020		−0.019		−0.019		−0.019	
	−0.018	−0.012	−0.025	−0.022	−0.029	−0.028	−0.031	−0.032	−0.033	−0.035	−0.045	−0.054

**Table A11:** Values *f*_fou,*i*,*j*_*,* [-] of the matrix **f**_fou_ of support displacement forms for *n* = 3, 4, 8, *k*_fou_ = 50, 100, 150, 200, 250 MN/m and *k*_fou_*-*rigid (elements *f*_fou,*j,j*_ (*i* = *j*) are given in bold).

*n*	*f*_fou,*i,j*_	*k*_fou_, MN/m					
		50	100	150	200	250	Rigid
3	*f*_fou,1,1_	**0**	**0**	**0**	**0**	**0**	**0**
	*f*_fou,1,2_	0	0	0	0	0	0
4	*f*_fou,1,1_	**−0.047**	**−0.026**	**−0.018**	**−0.014**	**−0.011**	**0**
	*f*_fou,2,1_	0.045	0.025	0.017	0.013	0.010	0
	*f*_fou,3,1_	−0.043	−0.024	−0.016	−0.013	−0.010	0
8	*f*_fou,1,1_	**−0.232**	**−0.147**	**−0.108**	**−0.086**	**−0.070**	**0**
	*f*_fou,2,1_	0.146	0.096	0.072	0.057	0.047	0
	*f*_fou,3,1_	−0.015	−0.016	−0.013	−0.011	−0.010	0
	*f*_fou,4,1_	−0.014	−0.007	−0.005	−0.004	−0.003	0
	*f*_fou,5,1_	−0.001	0.001	0.001	−0.004	0.001	0

**Table A12:** Values *f*_fou,*i*,*j*_, [-] of the matrix **f**_fou_ of support displacement forms for *n* = 16, *k*_fou_ = 50, 100, 150, 200, 250 MN/m and *k*_fou_*-*rigid (elements *f*_fou,*j,j*_ (*i* = *j*) are given in bold).

*i*	*k*_fou_, MN/m											
	50		100		150		200		250		Rigid	
	*f*_fou,*i*,1_	*f*_fou,*i*,2_	*f*_fou,*i*,1_	*f*_fou,*i*,2_	*f*_fou,*i*,1_	*f*_fou,*i*,2_	*f*_fou,*i*,1_	*f*_fou,*i*,2_	*f*_fou,*i*,1_	*f*_fou,*i*,2_	*f*_fou,*i*,1_	*f*_fou,*i*,2_
1	**−0.433**	0.193	**−0.315**	0.154	**−0.248**	0.128	**−0.205**	0.108	**−0.173**	0.093	**0**	0
2	0.187	**−0.501**	0.146	**−0.380**	0.119	**−0.306**	0.100	**−0.257**	0.085	**−0.219**	0	**0**
3	0.065	0.193	0.040	0.154	0.029	0.128	0.023	0.108	0.019	0.093	0	0
4	0.002	0.074	−0.005	0.051	−0.006	0.040	−0.006	0.032	−0.005	0.027	0	0
5	−0.020	0.001	−0.015	−0.006	−0.012	−0.007	−0.010	−0.007	−0.008	−0.006	0	0
6	−0.012	−0.007	−0.007	−0.006	−0.005	−0.005	−0.004	−0.004	−0.003	−0.003	0	0
7	−0.006	−0.012	−0.003	−0.008	−0.002	−0.006	−0.001	−0.005	−0.001	−0.004	0	0
8	0.000	0.000	0.000	0.002	0.000	0.002	0.000	0.002	0.000	0.002	0	0
9	0.001	0.000	0.001	0.000	0.000	0.000	0.000	0.000	0.000	0.000	0	0

**Table A13:** Values *f*_fou,*i*,*j*_, [-] of the matrix **f**_fou_ of support displacement forms for *n* = 32, *k*_fou_ = 50, 100, 150, 200, 250 MN/m and *k*_fou_*-*rigid (elements *f*_fou,*j,j*_ (*i* = *j*) are given in bold).

*i*	*k*_fou_, MN/m											
	50		100		150		200		250		Rigid	
	*f*_fou,*i*,1_		*f*_fou,*i*,1_		*f*_fou,*i*,1_		*f*_fou,*i*,1_		*f*_fou,*i*,1_		*f*_fou,*i*,1_	
	*f*_fou,*i*,2_	*f*_fou,*i*,3_	*f*_fou,*i*,2_	*f*_fou,*i*,3_	*f*_fou,*i*,2_	*f*_fou,*i*,3_	*f*_fou,*i*,2_	*f*_fou,*i*,3_	*f*_fou,*i*,2_	*f*_fou,*i*,3_	*f*_fou,*i*,2_	*f*_fou,*i*,3_
1	**−0.596**		**−0.476**		**−0.397**		**−0.342**		**−0.296**		**0**	
	0.175	0.106	0.153	0.088	0.134	0.074	0.118	0.065	0.105	0.057	0	0
2	0.172		0.148		0.128		0.112		0.098		0	
	**−0.632**	0.155	**−0.517**	0.140	**−0.439**	0.124	**−0.382**	0.111	**−0.334**	0.099	**0**	0
3	0.116		0.093		0.077		0.066		0.057		0	
	0.177	**−0.663**	0.158	**−0.548**	0.140	**−0.468**	0.124	**−0.409**	0.110	**−0.359**	0	**0**
4	0.000		0.000		0.000		0.000		0.000		0	
	0.000	0.155	0.000	0.140	0.000	0.124	0.000	0.111	0.000	0.099	0	0
5	0.035		0.022		0.016		0.013		0.011		0	
	0.081	0.106	0.063	0.087	0.052	0.074	0.044	0.064	0.038	0.056	0	0
6	0.009		0.002		0.000		−0.001		−0.001		0	
	0.042	0.062	0.029	0.047	0.023	0.038	0.019	0.032	0.016	0.027	0	0
7	−0.005		−0.008		−0.007		−0.007		−0.006		0	
	0.015	0.030	0.008	0.019	0.005	0.014	0.003	0.011	0.002	0.009	0	0
8	0.000		0.000		0.000		0.000		0.000		0	
	0.000	0.000	0.000	0.000	0.000	0.000	0.000	0.000	0.000	0.000	0	0
9	−0.012		−0.008		−0.006		−0.005		−0.004			
	−0.011	−0.007	−0.011	−0.009	−0.009	−0.009	−0.008	−0.008	−0.007	−0.008	0	0

**Table A14:** Work done by the jacks during the forced piston extension in all the jacks by *u*_ext_ = 1 mm and mean displacement of the object *u*_obj-mean_ for *n* = 3, 4, 8, 16 and 32, *k*_fou_ = 50, 100, 150, 200, 250 MN/m and *k*_fou_*-*rigid.

*n*	*k*_fou_, MN/m	50	100	150	200	250	Rigid
3	*W*, kJ	1.347	1.347	1.347	1.347	1.347	1.347
	*u*_obj-mean_, mm	1	1	1	1	1	1
	*W*/*u*_obj-mean_ kJ/mm	1.347	1.347	1.347	1.347	1.347	1.347
4	*W*, kJ	1.347	1.347	1.347	1.347	1.347	1.347
	*u*_obj-mean_, mm	1.031	1.035	1.035	1.036	1.035	1.038
	*W*/*u*_obj-mean_ kJ/mm	1.306	1.301	1.301	1.300	1.301	1.297
8	*W*, kJ	1.347	1.347	1.347	1.347	1.347	1.347
	*u*_obj-mean_, mm	1.151	1.193	1.212	1.226	1.233	1.272
	*W*/*u*_obj-mean_ kJ/mm	1.170	1.129	1.111	1.098	1.092	1.059
16	*W*, kJ	1.366	1.362	1.359	1.357	1.356	1.350
	*u*_obj-mean_, mm	1.307	1.461	1.547	1.606	1.657	1.923
	*W*/*u*_obj-mean_ kJ/mm	1.045	0.932	0.879	0.845	0.818	0.702
32	*W*, kJ	1.373	1.387	1.396	1.403	1.409	1.448
	*u*_obj-mean_, mm	1.414	1.677	1.857	1.995	2.106	2.911
	*W*/*u*_obj-mean_ kJ/mm	0.971	0.827	0.752	0.703	0.669	0.497

**Table A15:** Force *Q_g,_* in the jacks depending on the *n* number of the jacks and stiffness *k*_fou_ of jack supports.

*n*	*Q_g,i_*	*k*_fou_, MN/m					
		50	100	150	200	250	rigid
3	*Q_g_*_,1_, kN	431.30	431.30	431.30	431.30	431.30	431.30
	*Q_g_*_,2_, kN	457.80	457.80	457.80	457.80	457.80	457.80
4	*Q_g_*_,1_, kN	336.72	336.72	336.72	336.72	336.72	336.72
8	*Q_g_*_,1_, kN	168.35	168.35	168.35	168.35	168.35	168.35
16	*Q_g_*_,1_, kN	66.65	60.05	56.59	54.46	52.90	45.50
	*Q_g_*_,2_, kN	101.69	108.28	111.74	113.86	115.43	122.82
32	*Q_g_*_,1_, kN	21.33	13.49	9.37	6.83	4.96	−3.89
	*Q_g_*_,2_, kN	46.21	47.83	48.69	49.22	49.62	51.51
	*Q_g_*_,3_, kN	54.60	59.27	61.70	63.19	64.28	69.42
